# Randomized cross-over study investigating the tolerability and side effects of an intra-oral air-cooling device compared to ice in healthy volunteers

**DOI:** 10.1007/s12032-022-01932-4

**Published:** 2022-12-23

**Authors:** C. Blacker, R. S. Bejhed, P. Frykholm, G. Ljungman

**Affiliations:** 1 Department of Women’s and Children’s Health, Uppsala University, SE-751 85 Uppsala, Sweden; 2 Department of Surgical Sciences, Section of Anaesthesia and Intensive Care Medicine, Uppsala University, SE-751 85 Uppsala, Sweden

**Keywords:** Innovative medical technology, Intraoral air-cooling device, Oral cryotherapy, Oral mucositis

## Abstract

Oral cryotherapy (OC) is a common preventive treatment of oral mucositis (OM) and is recommended in international guidelines. Ice and air OC have previously been shown to result in temperature reductions of 8.1–12.9 °C, and 14.5 °C, respectively, in healthy volunteers. However, no direct comparison between these two modalities has been performed. The primary aim was to investigate the tolerability and side effects of air OC using an intra-oral air-cooling (IOAC) device compared with ice OC. The secondary aim was to evaluate the temperature reduction in the mouth for the two respective methods. Cross-over study with randomization to order of treatment, in 15 healthy volunteers. We evaluated the self-reported intensity, frequency, and discomfort for 13 pre-defined side effects used in previous studies. All participants were able to complete both OC sessions, although one participant required reduced airflow in the air OC arm. The subjects reported more discomfort from being cold, having sensitive teeth, and numbness in the ice OC group, while they reported more discomfort from swallowing when subjected to air OC. No significant difference in the median temperature reduction was detected in the two modalities, except for the dorsal posterior part of the tongue where temperature reduction was larger in the ice OC group. We found that oral cooling using a new IOAC device was tolerated and seems to be safe in healthy volunteers.

## Introduction

Oral cryotherapy (OC) is a recommended preventive treatment of oral mucositis (OM) in international guidelines [1–4]. OC is usually achieved by letting ice chips, slushies, or popsicles melt in the mouth during chemotherapy, and is a cost-effective and safe treatment with few side effects [5].

OM is a common side effect of chemotherapy and radiotherapy in adults and children presenting as erythematous sores and ulcerations in the oral mucosa [3, 5–7]. OM causes pain, nutritional difficulties, and increases the risk for infections, and is considered to be one of the most debilitating side effect according to patients [9–14]. OM can be a dose-limiting factor resulting in chemotherapy dose reduction and increased healthcare costs [12, 15]. The incidence rate of OM is around 20–40% for patients receiving standard chemotherapy regimens, while for radiotherapy towards head and neck, it is close to 100%, and for patients receiving hematopoietic stem cell transplant (HSCT), it is close to 80% [6, 10, 16, 17]. Risk factors for developing OM vary with treatment regimens, genetic factors, age, and smoking, as well as previous episodes of OM [7, 16–18].

OC is suggested to prevent OM by local cooling causing vasoconstriction in the tissue, which leads to less chemotherapy exposure and a reduction of the metabolism of the basal epithelial cells resulting in a lower toxicity of the chemotherapeutic agent and, therefore, less tissue damage [8, 19, 20]. Treatment times of 30 to 60 min have been evaluated in randomized clinical trials [19, 21–23]. The reported temperature reduction of the oral mucosa was 8.1–12.9 °C using ice chips and 14.5 °C for air OC [24–26]. However, no direct comparison between these two modalities has been performed. To address this lack of data, we undertook a randomized cross-over study in healthy volunteers comparing the two methods.

The primary aim of this study was to investigate the tolerability and side effects of an intra-oral air-cooling (IOAC) device compared to OC with ice. The secondary aim was to evaluate the temperature reduction of the mouth for the two respective methods.

## Subjects and methods

This was a cross-over study, randomized with regard to order of treatments. The study was approved by the Swedish Ethical Review Authority (Dnr 2021–01,485).

### Participants

Measurements were performed on fifteen healthy volunteers, recruited by open advertisement at the Department of Anaesthesia and Intensive Care at Uppsala University Hospital, Uppsala, Sweden (*n* = 1) and a Facebook group (*n* = 14) which is dedicated mainly to medical students at Uppsala University. The study recruited more women (*n* = 9), and the mean age was 25.9 years (SD 4.9, range 20 to 40) (Table 1).

**Table 1:** Subject characteristics

Age in years	Mean (SD)	25.9 (± 4.9)
	Range	20–40
Gender	Female	9
	Male	6
Health problems	No	15
Dental problems	No	15
Sensitive teeth	Yes	6
How often do you have sensitive teeth	Rarely	5
	Sometimes	1
	Often	0

*SD* standard deviation

### Methods

Tolerability was defined as the participant being able to maintain the OC protocol for > 90% of the total intended treatment time. Short interruptions were allowed in both study arms.

A questionnaire based on a modified numeric rating scale (NRS) rating the different side effects was created. Each side effect was quantified from intensity, frequency, and discomfort perspectives, respectively. Thus, zero represented no intensity at all, never experiencing the side effect or no discomfort in the first, second, and third categories, respectively. In analogy, the number 10 represented maximum intensity, continuous experience of the side effect, or maximum discomfort in the three respective categories. The choice of parameters was based on previous publications [25–27]. In order to make the grading more comprehensible, it was decided to categorize the grade of side effects as low (median score 0–4), moderate (median score > 4–7), and severe (median score > 7–10).

**Fig. 1:**
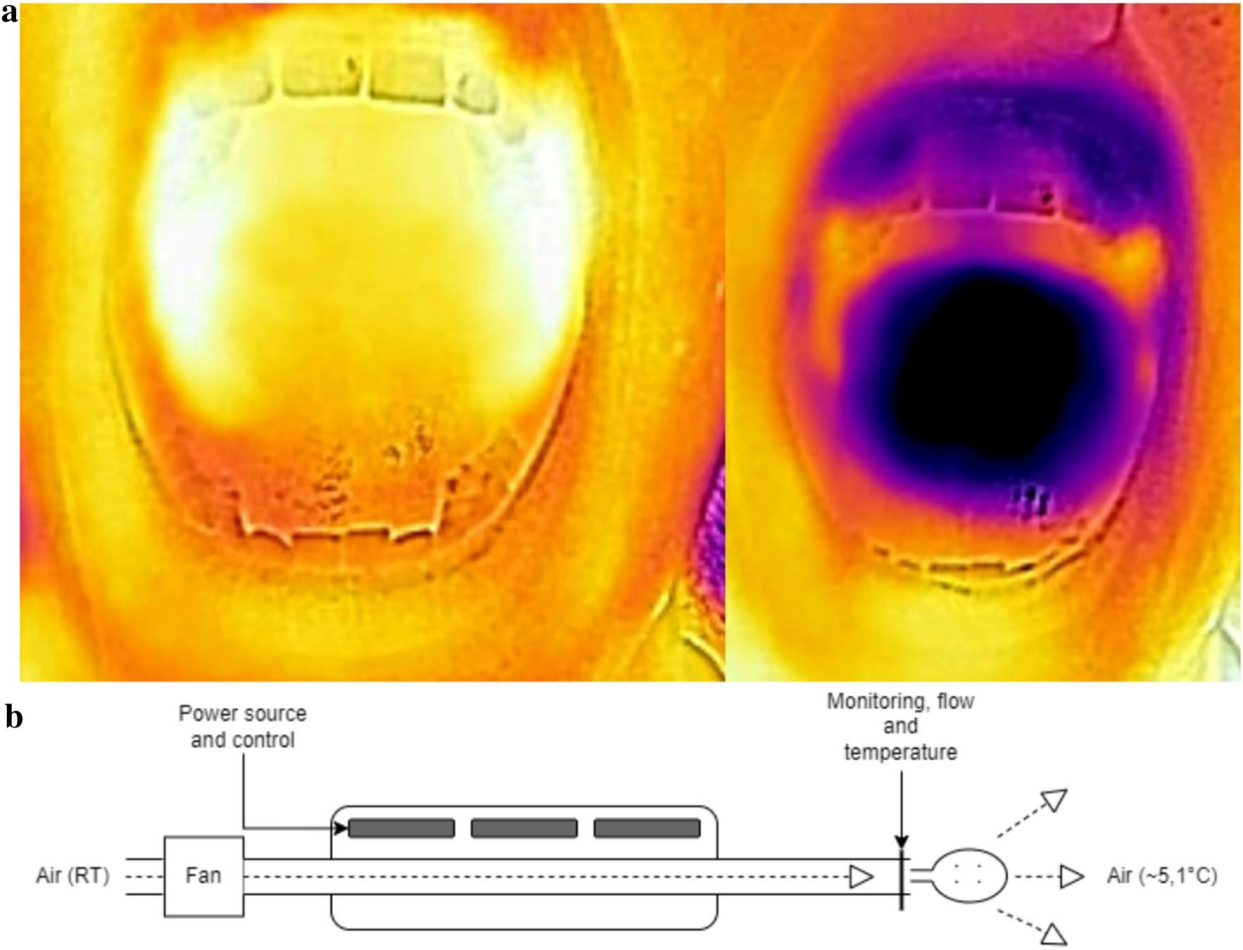
**a** Example of digital thermal images of the oral mucosa before and after the intervention. The left image with white/yellow color range areas represents the highest temperatures. The right image with black/purple color range represents the sites of lowest temperatures. **b** Schematic drawing of the IOAC device. *RT* room temperature

Temperature measurements were obtained using a FLIR® C digital thermal imaging camera. All participants were placed seated in a position 25 cm from the camera. The specifications of the thermal camera used for this study were as follows: minimal focus distance of 0.15 m, accuracy 0.01 °C, thermal sensitivity < 0.10 °C, and penetration of 4.5–6.5 mm (spectral range 7.5–14 µm) of the outer mucosal surface [28, 29]. The temperature differences on the output JPG images are visualized as color gradients based on a choosable palette. Using a rainbow color palette, the colors on the image ranges from white/yellow, representing the highest temperature, to black, representing the lowest temperature. Images were downloaded to a computer using the FLIR tools + software (Version 6.4.18031.1003) for data analysis (Fig. 1a).

### The air-cooling device

We used a novel IOAC that was described for the first time in a previous study [24]. It consists of a cooling unit, a cooling delivery system, and a mouthpiece (Fig. 1b). The average temperature of the cooled air was 5.1 °C, measured at the mouthpiece.

### Experimental procedure

Before participating in the study, each participant had received written information about the study and was asked to schedule two separate appointments that would have to be at least one day apart. Each participant was asked not to consume tobacco, alcohol, caffeinated drinks, medications, or food 60 min prior to the study protocol. Informed consent was obtained at the first appointment after additional verbal information about the study had been provided. Basic demographic and health data were obtained from the participants to ensure that they fulfilled the inclusion criteria (age 18–65 years, speak, and understand Swedish or English fluently, and being healthy) and had none of the exclusion criteria (diseases requiring medical care or dental problems requiring care from a dentist). Afterwards, the volunteers were placed in a room for acclimatization with an average temperature of 23.7 °C for 30 min before initiating the experiment. At this point, the participants were randomized to initiate the experiment with either ice or air OC. Randomization was based on a simple coin toss.

Baseline images of the oral mucosa were obtained from the right and left buccal mucosa, upper and lower lips, anterior, posterior, and ventral aspects of the tongue and the hard palate. During the experiment, output data regarding temperature and flow were continuously collected from the IOAC device.

If the participant was randomized to air OC, the experiment was initiated with a five-minute test phase to test the IOAC, and in case, any adjustments were needed. Thereafter, the experiment continued through 60 min and a new set of images was collected of the above-mentioned areas.

If the participant was randomized to ice OC, the same procedure with baseline images was performed, and thereafter, a bucket of crushed ice was made available. Each participant was encouraged to keep the ice in the mouth for as long as they could (maximum 60 min). The participants were free to swallow or spit out the ice water. After this, a new set of images was collected of the above-mentioned areas. The average amount of ice consumed was 256 g, which was calculated by measuring the quantity of ice before initiating and after completing the experiment. After completing the experiment, the participants were asked to answer a questionnaire.

During the participant’s second session, they would repeat the same procedure but with the other type of OC, and the same questionnaire was given after they completed the experiment. After approximately one month, the participants were asked if they would consider participating in the experiment again, which OC treatment type they preferred, and in a hypothetical scenario of OC treatment for four hours (assuming equal effectiveness), which type they would prefer.

### Statistics

No formal power analysis was performed since it was not an effect study, but rather a proof-of-concept study. The IBM SPSS Statistics (version 28.0.1.0 (142)) was used for statistical analysis. Shapiro–Wilk’s test (*p* > 0.05), visual inspection of histograms, and normal Q-Q plots indicated that most temperature reduction variables were approximately normally distributed, but some were not. Due to the small study population and mixed normality test results, it was decided to treat all data as non-normally distributed and a non-parametric Wilcoxon-Signed Ranks Test was used. Significance level was set to *p* < 0.01 due to multiple testing and relatively few observations.

## Results

All participants reported being healthy and had no dental conditions requiring dentist intervention. Nine self-reported not having sensitive teeth, 5 rarely had sensitive teeth, and 1 had sensitive teeth sometimes (Table 1).

### Sample characteristics

All participants (*n* = 15) were able to complete both OC sessions, although one participant required reduced airflow in the air OC arm. The most common side effects for ice OC were feeling cold (*n* = 15), numbness (*n* = 14), or pain in the mouth (*n* = 11). The most common side effects for air OC were increased saliva production (*n* = 14), swallowing difficulties (*n* = 13), mouth dryness (*n* = 12), and feeling cold in the mouth (*n* = 12) (Table 2).

**Table 2:** Table of participants’ number of reported side effects (*N* = 15)

	Intervention	
	Ice	Air
	N	N
Side effect		
Cold	15	12
Numbness	14	5
Bad taste	7	7
Headache	4	5
Sensitive teeth	10	5
Intraoral pain	11	7
Difficulties keeping mouthpiece in the mouth	N/A	9
Nausea	6	6
Difficulties swallowing	6	13
Bad fit	N/A	8
Jaw tension	3	7
Hypersalivation	8	13
Mouth dryness	4	12

We evaluated self-reported scores for intensity, frequency, and discomfort (median [IQR]) for the various side effects. Each of the side effects was thereafter ranked according to grade.

**Fig. 2:**
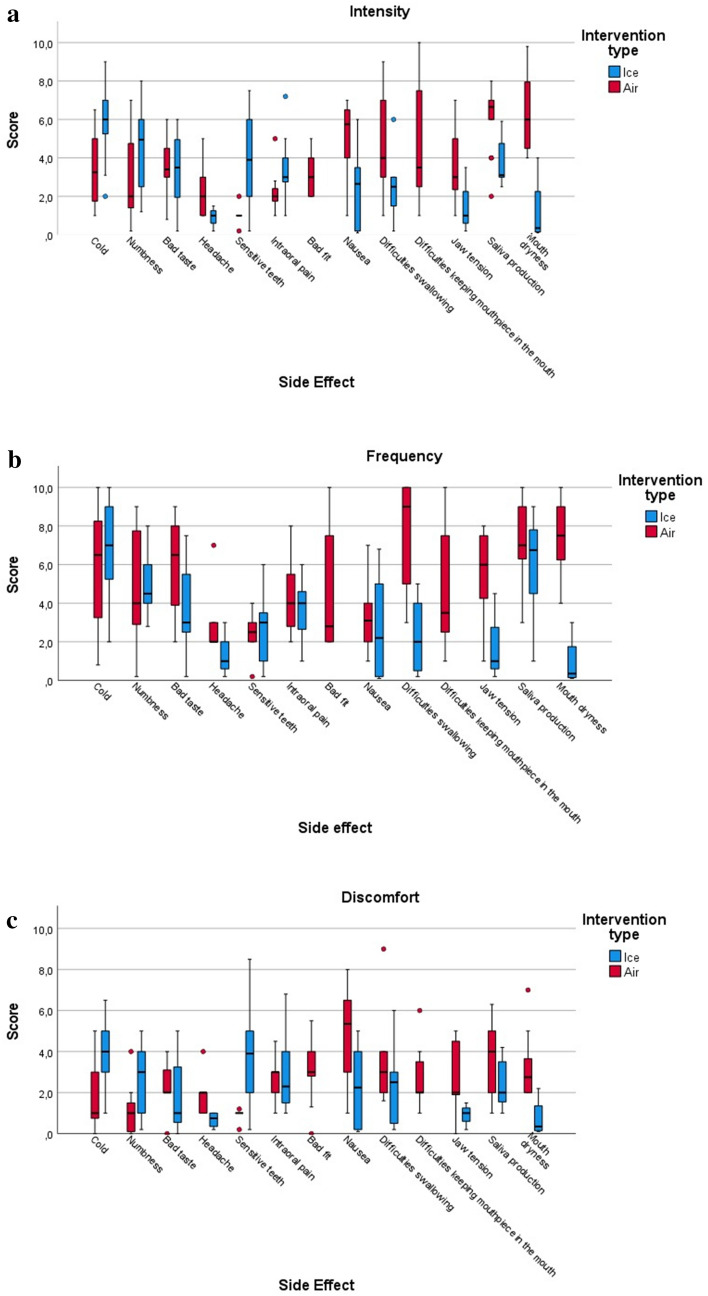
Cluster Boxplot of the **a** Intensity, **b** Frequency, and **c** Discomfort of Side effects for the two interventions. Black line in colored area represents the median, colored area represents IQR, whiskers represent range excluding outliers, circles represent outliers

We found higher intensity scores for ice OC compared to air OC: feeling cold in the mouth (6.0 [2.0] vs. 3.3 [3.3]), numbness (5.0 [3.5] vs. 2.0 [5.5]), and sensitive teeth (3.9 [2.0] vs. 1.0 [0.0]). In contrast, we found higher scores for hypersalivation (6.7 [1] vs. 6.7 [1.0]), dry mouth (6 [3.5] vs. 0.4 [2.1]), and difficulty swallowing (4 [4] vs. 4.0 [4.0]) for air OC (Fig. 2a & Table 3).

**Table 3:** Table of participants’ scored side effects (*N* = 15)

	Ice OC				Air OC				
	Mean	Median	IQR	Score	Mean	Median	IQR	Score	*p* value
Side effect									
Cold intensity	5.9	6.0	2.0	Moderate	3.5	3.3	3.3	Low	< .001*
Cold frequency	6.7	7.0	4.5	Severe	5.9	6.5	5.0	Moderate	.08
Cold discomfort	4.0	4.0	2.0	Moderate	2.1	1.5	2.5	Low	< .001*
Numbness intensity	4.7	5.0	3.5	Moderate	3.1	2.0	5.5	Low	.002*
Numbness frequency	4.9	4.5	2.0	Moderate	4.9	4.0	6.0	Low	.008*
Numbness discomfort	2.6	3.0	3.0	Low	1.6	1.0	1.0	Low	.003*
Bad taste intensity	3.4	3.5	5.0	Low	3.6	3.4	1.5	Low	.760
Bad taste frequency	3.8	3.0	5.0	Low	6.0	6.5	4.1	Moderate	.185
Bad taste discomfort	1.9	1.0	3.3	Low	2.6	2.0	1.2	Low	.400
Headache intensity	.9	1.0	.7	Low	2.4	2.0	2.0	Low	.176
Headache frequency	1.3	1.0	1.4	Low	3.2	2.0	1.0	Low	.168
Headache discomfort	.7	.8	.7	Low	2.0	2.0	1.0	Low	.112
Sensitive teeth intensity	3.9	3.9	2.0	Low	1.0	1.0	.0	Low	.008*
Sensitive teeth frequency	2.8	3.0	2.5	Low	2.3	2.5	1.0	Low	.049
Sensitive teeth discomfort	3.9	3.9	3.0	Low	.9	1.0	.0	Low	.008*
Intraoral pain intensity	3.5	3.0	1.5	Low	2.3	2.0	1.3	Low	.023
Intraoral pain frequency	3.8	4.0	2.7	Low	4.4	4.0	3.4	Low	.327
Intraoral pain discomfort	3.0	2.3	3.0	Low	2.6	3.0	1.5	Low	.238
Difficulties keeping mouthpiece in the mouth intensity	N/A				3.3	3.0	2.0	Low	
Difficulties keeping mouthpiece in the mouth frequency	N/A				5.0	2.8	5.5	Low	
Difficulties keeping mouthpiece in the mouth discomfort	N/A				3.1	3.0	1.2	Low	
Nausea intensity	2.5	3.0	2.8	Low	5.0	5.8	2.5	Moderate	.028
Nausea frequency	2.8	2.2	4.8	Low	3.4	3.1	2.0	Low	.866
Nausea discomfort	2.3	2.3	3.8	Low	4.9	5.4	3.5	Moderate	.028
Difficulties swallowing intensity	2.6	2.5	1.5	Low	5.0	4.0	4.0	Low	.001*
Difficulties swallowing frequency	2.3	2.0	3.5	Low	7.4	9.0	5.0	Severe	.001*
Difficulties swallowing discomfort	2.2	2.5	2.7	Low	3.2	3.0	2.0	Low	.005*
Bad fit intensity	N/A				4.8	3.5	5.0	Low	
Bad fit discomfort	N/A				2.8	2.0	1.5	Low	
Jaw tension intensity	1.6	1.0	3.3	Low	3.6	3.0	2.7	Low	.018
Jaw tension frequency	1.9	1.0	4.3	Low	5.6	6.0	3.3	Moderate	.018
Jaw tension discomfort	.9	1.0	1.3	Low	3.1	2.0	3.0	Low	.017
Hypersalivation intensity	3.8	3.1	1.8	Low	6.1	6.7	1.0	Moderate	.002*
Hypersalivation frequency	6.0	6.8	3.3	Moderate	7.4	7.0	2.7	Moderate	.004*
Hypersalivation discomfort	2.4	2.0	2.0	Low	3.8	4.0	3.0	Low	.011
Mouth dryness intensity	1.2	.4	2.1	Low	6.2	6.0	3.5	Moderate	.003*
Mouth dryness frequency	1.0	.4	1.6	Low	7.5	7.5	2.8	Severe	.002*
Mouth dryness discomfort	.8	.4	1.2	Low	3.2	2.8	1.7	Low	.006*

Side effects were graded as low (median score 0–4), moderate (median score > 4–7), and severe (median score > 7–10). *IQR* inter quartile range, *N/A* not applicable

*p value < 0.01

Regarding frequency, the higher score in the ice OC group was numbness (4.5 [2] vs. 4.0 [6.0]). In contrast, difficulty swallowing (9 [5] vs. 9.0 [5.0]), mouth dryness (7.5 [2.8] vs. 6.8 [3.3]), and increased saliva production (7 [2.7] vs. 3.1 [1.8]) were more frequently experienced in the air OC group (Fig. 2b & Table 3).

Finally, we found higher discomfort scores from being cold (4 [2] vs 1.5 [2.5]), sensitive teeth (3.9 [3] vs. 1 [0]), and numbness (3 [3] vs 1 [1]) in the ice OC compared to air OC. In contrast, the participants reported higher discomfort scores from difficulty in swallowing (3 [2] vs 2.5 [2.7]) when using air OC (Fig. 2c and Table 3).

One participant was not able to utilize air OC per protocol and needed reduced air flow levels to tolerate the treatment. For this reason, this participant was removed from subsequent analyses. After this, the median temperature reduction was 14.1 ± 4.3 °C and 12.0 ± 3.5 °C in the oral mucosa for ice and air OC, respectively. This difference was not significant; the only location in the mouth where we found a significant difference in temperature reduction was the dorsal posterior part of the tongue for ice OC 24.3 ± 7.1 °C compared to air OC 16.4 ± 4.2 °C (Table 4).

**Table 4:** Median and mean temperature reduction in the oral mucosa, without one participant (*N* = 14) due to non-adherence to protocol, with different interventions

	Intervention								
	Ice				Air				
	Mean	Median	IQR	Range	Mean	Median	IQR	Range	*p* value
Site									
All surfaces	13.9	14.1	4.29	3.5–18.8	11.9	12,0	3.53	5.5–15.9	0.084
Tongue dorsum anterior	15.6	13.5	10.1	5.7–28.5	14.0	14.6	6.4	6.8–20.3	0.778
Tongue dorsum posterior	22.8	24.3	7.1	9.6–29.6	16.6	16.4	4.2	8.4–23.1	0.008*
Hard palate	10.7	10.9	2.7	1.4–17.4	13.5	12.4	7.2	6.9–20.3	0.096
Soft palate	14.4	14.5	7.6	− 1.3–24.4	14.1	14.1	5.1	8.4–17.6	0.925
Buccal left	18.0	18.7	4.9	3.8–26.9	16.3	15.6	3.6	6.5–26.3	0.363
Buccal right	16.6	17.3	2.6	1.5–26.5	14.6	14.8	1.4	6.6–19.5	0.177
Upper lip	13.0	14.9	9.2	5.5–19.4	9.6	9.5	3.3	17.4	0.064
Lower lip	8.3	8.5	6.8	2.6–12.8	6.4	6.3	4,0	13.5	0.158
Tongue ventral	9.0	9.0	4.7	0.8–16.0	5.8	6.3	3.7	9.1	0.011
Mouth bottom	12.4	12.5	8.3	4.2–20.1	8,0	8,0	3.4	15.6	0.028

*p < 0.01

When participants were asked if they would consider participating in the experiment again, 13 responded that they would and 2 were indecisive. Six participants said they preferred air OC, four preferred ice OC, and one had equal preferences for both. Four individuals could not give a preference regarding preferred type of OC. In the hypothetical scenario of four hours of OC, six said that they would prefer air OC, four would prefer ice OC, one indicated equal preferences for both, and four were indecisive.

## Discussion

All participants completed the study protocol; both ice and air OC met the pre-defined tolerability criterion. Tolerability was similar or better than what has been previously reported for ice OC [24–26]. The most common side effects for ice OC were feeling cold, numbness, or pain in the mouth, probably related to the cooling effect of ice. Similar results were reported by Walladbegi et al. [26]. Swallowing difficulties and poor fit seem to be common side effects of the IOAC device and could be related to device design [26, 27]. In addition, increased salivation and dry mouth seem to affect participants more often with the IOAC device than with ice OC. This is probably related to the air flow, which we have previously reported [24].

To the best of our knowledge, this is the first study to measure and compare the side effects of two different OC modalities in healthy individuals. The participants’ evaluation of the intensity of side effects for ice OC was of moderate to low grade (cold, numbness and sensitive teeth), and similarly, that for air OC was of moderate to low grade (hypersalivation, dry mouth and difficult swallowing), indicating an acceptable intensity level of the experienced side effect. In contrast, the frequency for some of these side effects was of low grade for ice OC (numbness), while severe to moderate grade for air OC (difficulty swallowing, dry mouth and hypersalivation). These differences in the frequency can be explained by the IOAC device being continuously in the month, thereby making for a more frequent perception of these side effects, while side effects of ice OC will remain only for as long as the ice slowly melts in the mouth. Reassuringly, when the participants were asked to evaluate the total discomfort of the various side effects, the result was low grade in both the ice OC (cold, sensitive teeth, and numbness) and air OC arms (difficulties swallowing).

With regard to the temperature reduction in the oral mucosa, the only significant result was observed on the dorsal part of the tongue, where ice OC resulted in a larger temperature reduction. The temperature reduction in the ice OC group in our study was larger than in previously reported studies, and our air OC temperature reduction was also similar or larger than the ice temperature reduction previously reported [25–27]. This indicates that air OC has the capacity to reduce the temperature in the oral mucosa to a degree that is clinically relevant. Larger temperature reductions accomplished by air OC have been demonstrated previously by our group [24]. This suggests that if the treatment is tolerable at the intended air flows, temperature reduction in the oral mucosa achieved by air OC is similar to that of ice OC.

The strengths of this study lie in the experimental nature of the cross-over trial design making each participant their own control in combination with the randomization of the order of treatments to eliminate order effect. We focused on collecting only a few data points, which were continuously monitored, and the study was performed in a controlled environment. The same person collected the raw data and evaluated the images for all subjects. This is on one hand a strength because it provides for a more consistent analysis, but on the other hand presents a limitation due to the risk of observer bias. We allowed for a minimum of one day wash-out to restore oral mucosa temperature and sensibility and to enable the participants to answer the questionnaires with some time passing in between. Furthermore, the follow-up questions regarding willingness to participate in a similar study again took place a month later, in order to allow the participants plenty of time for reflection, and to monitor the development of any late side effects.

A limitation of the study was that the power was low due to few participants and a large number of measurements; however, we considered the sample size to be adequate for a proof-of-concept study. Likewise, some of the participants indicated that they would take part in a similar study and more of them preferred air OC compared to ice OC in a hypothetical scenario of 4 h of treatment, but due to the small sample size, no firm conclusion can be made from this data. Another limitation is the use of healthy volunteers who are not at risk of developing OM, which might increase or decrease motivation to fulfill the intended treatment, resulting in worse or better tolerability than the target population would have demonstrated. However, for ethical reasons, it was necessary to start with a study in volunteers. Finally, it was for obvious reasons not feasible to blind the participants to the treatment modality.

## Conclusion

We found that oral cooling using a new IOAC device was tolerated and seems to be safe in healthy volunteers. Further studies are needed to explore its efficacy for preventing oral mucositis in patients receiving chemotherapy.
